# Association between Serum Interleukin-17A Level and High-Altitude Deacclimatization Syndrome

**DOI:** 10.1155/2016/1732352

**Published:** 2016-04-14

**Authors:** Binfeng He, Hongli Li, Mingdong Hu, Weijie Dong, Zhenghua Wei, Jin Li, Wei Yao, Xiaolan Guo

**Affiliations:** ^1^Institute of Respiratory Diseases, Xinqiao Hospital, The Third Military Medical University, Chongqing 400037, China; ^2^Department of Laboratory Medicine, North Sichuan Medical College, Nanchong, Sichuan 637000, China; ^3^Translational Medicine Research Center, North Sichuan Medical College, Nanchong, Sichuan 637000, China

## Abstract

High-altitude deacclimatization syndrome (HADAS) is emerging as a severe public health issue that threatens the quality of life of individuals who return to lower altitude from high altitude. In this study, we measured serum levels of SOD, MDA, IL-17A, IL-10, TNF-*α*, and HADAS score in HADAS subjects at baseline and 50th and 100th days and to evaluate the relationship between interleukins, including IL-17A, and HADAS. Our data showed that and the serum IL-17A levels and HADAS score decreased over time in the HADAS group, and serum IL-17A levels were significantly higher in the HADAS group at baseline and 50th day compared with controls (*p* < 0.05). Furthermore, baseline serum levels of MDA and TNF-*α* were significantly higher, while SOD and IL-10 levels were lower in HADAS subjects compared with controls (*p* < 0.05). It is interesting that serum levels of IL-17A were clearly interrelated with HADAS incidence and severity (*p* < 0.05). ROC curve analysis showed that combined serum IL-17A and IL-10 levels were a better predictor of HADAS incidence than serum levels of IL-17A or IL-10 alone. These data suggest that serum levels of IL-17A are a novel predictive index of HADAS.

## 1. Introduction

When people originally from a lower altitude descend to sea level or lower altitude after high-altitude/hypoxia acclimatization, then they lose hypoxia tolerance and physiological adjustments. In addition, they experience changes in hemoglobin and hormone levels over time, and this is known as high-altitude deacclimatization (HADA) [1]. This physiological process has been documented in explorers [2], athletes [3], military personnel [4], and workers in high-altitude mines [5]. Following recent dramatic economic growth in plateau regions of China, such as Tibet, Qinghai, and Xinjiang, tens of millions of lower altitude individuals temporarily migrated to high-altitude regions for work, and then they returned to lower altitudes after they finished their work each year. When the workers returned to lower altitudes, most of them were in the process of HADA and suffered from physical discomfort and symptoms. A previous study has shown that individuals suffering from HADA experienced symptoms such as sleepiness, insomnia, unresponsiveness, memory loss, fidgetiness, headache, throat pain or discomfort, coughing, sputum, chest tightness, becoming flustered, increased appetite, decreased appetite, diarrhea, abdominal distention, abdominal pain, lumbago, and arthralgia [6]. These symptoms are characteristic and are usually referred to as HADA syndrome (HADAS), which affects the quality of life of these individuals [7, 8]. Our previous and other studies showed that HADAS subjects suffered a series of clinical symptoms, which could last for many years [6, 9–11]. Therefore, HADAS has been a public health issue in China and in other countries.

In our previous study [6], we found that subjects who suffered from HADAS experienced hypoxia/reoxygenation (H/R). The subjects lived in hypoxic conditions and then quickly returned to normoxic environments, and the levels of PaO_2_ and SO_2_ in HADAS subjects rapidly increased from 81.58 hPa and 87.31% to 125.84 hPa and 96.78%, respectively. Evidence from studies showed that H/R induced oxidative stress and production of reactive oxygen species (ROS) [12, 13] and resulted in damage to tissue or cells [14]. Superoxide dismutase (SOD) plays an important role in removal of excess free radicals in humans experiencing oxidative stress [15, 16]. Malondialdehyde (MDA) is a product of lipid peroxidation, which occurs when unsaturated lipids are exposed to oxygen [15, 16]. Elevation of MDA levels leads to increased oxidative stress and oxidative-mediated damage [15, 16]. Zhou et al. [17] showed that serum SOD levels were elevated and MDA levels were decreased when subjects returned to lower altitudes upon short-term exposure to high altitudes. However, the roles of SOD and MDA in HADAS are unknown.

Research has shown that H/R could increase generation of proinflammatory mediators, such as tumor necrosis factor-*α* (TNF-*α*) [18] and interleukin- (IL-) 17A [19], and suppress levels of anti-inflammatory factor IL-10 [20], and then it induced apoptosis and damage to cells and tissues [21, 22]. In our previous study, we showed that a systemic inflammatory response and myocardial injury were observed in HADAS subjects 3 d after returning to a lower altitude [6]. However, serum levels of IL-17A, IL-10, and TNF-*α* in HADAS subjects as well as correlations between these factors and occurrence rate and progression of HADAS are not clear.

In this study, we evaluated the HADAS score and measured serum levels of SOD, MDA, IL-17A, TNF-*α*, and IL-10 in HADAS and control groups. We then analyzed the correlation of serum levels of SOD, MDA, IL-17A, TNF-*α*, and IL-10 with HADAS occurrence and severity.

## 2. Methods

### 2.1. Subjects and General Protocols

Sixty-seven healthy male subjects (25.1 ± 7.6 years old) from Chongqing (180 m) had worked in Lhasa (3650 m) for about 8 months and then returned to Chongqing by airplane. After they returned, all subjects had been diagnosed with mild-to-moderate HADAS according to relevant diagnostic and scoring criteria, and they were evaluated for HADAS at 3 d (baseline), 50 d, and 100 d. They were considered the HADAS group. In addition, 41 healthy male subjects (24.8 ± 8.1 years old) who had always lived in Chongqing served as the control group. The 2 groups were not significantly different in age (*p* > 0.05) (Table 1). All subjects provided written informed consent. This study was approved by the Medical Ethical Committee of the Second Affiliated Hospital, Third Military Medical University.

### 2.2. Diagnostic and Scoring Criteria for HADAS

Subjects had been diagnosed with HADAS according to relevant diagnostic and scoring criteria [6]. Briefly, adult individuals who were less than 60 years old returned to a lower altitude from a higher altitude where they had worked for 4–12 months. They suffered from 3 or more of the following symptoms: fatigue, sleepiness, insomnia, unresponsiveness, memory loss, fidgetiness, headache, and throat pain or discomfort. The principal exclusion criteria included symptoms directly attributable to primary diseases affecting the cardiovascular, respiratory, nervous, urinary, and hematological systems, cancer or leukemia, and a recent history of influenza, upper respiratory tract infection, infectious diarrhea, or similar symptoms. HADAS symptom scores (HADAS scores) were evaluated according to the scoring criteria for HADAS. Scores from 6 to 15 indicated a mild reaction, and scores from 16 to 25 indicated a moderate reaction.

### 2.3. Collection and Analysis of Blood Samples

Morning fasting venous blood (3 mL) was collected, centrifuged at 4000 r/min for 10 min to separate serum, and stored at −80°C before assay. Human serum IL-17A, TNF-*α*, and IL-10 ELISA kits were purchased from R&D Systems (Abingdon, UK). Serum IL-17A, TNF-*α*, and IL-10 levels were detected according to the manufacturer's instructions. Optical density at 450 nm was measured with a spectrophotometer.

SOD and MDA assay kits were purchased from the Nanjing Jiancheng Bioengineering Institute (Nanjing, China). Serum SOD levels were measured according to the manufacturer's instructions. Briefly, the serum of subjects was mixed with the reagents and incubated for 40 min at 37°C. After the reaction, absorbance at 560 nm was monitored using a spectrophotometer. The samples were then mixed with trichloroacetic acid and incubated for 40 min at 95°C. The absorbance of each sample was measured at 532 nm with a spectrophotometer. MDA concentration was calculated according to the formula provided in the protocol.

### 2.4. Statistical Analysis

SPSS 15.0 for Windows was used for statistical analysis. All data are presented as the arithmetic mean values (SD) or mean (SE, 95% CI). The changes in HADAS scores and levels of serum factors between HADAS and control group were examined across the study time course with a linear mixed effects modeling approach, and age and times were considered as covariates. Correlations of scores, SOD, MDA, IL-17A, IL-10, TNF-*α*, and ages of HADAS subjects at baseline were analyzed using Pearson's correlation test. The effect of cytokine with HADAS occurrence and severity was analyzed via covariance adjusted logistic regression analysis, respectively, and age was considered as covariance. Based on logistic regression analysis, significant variables associated with HADAS occurrence were incorporated into a multivariate logistic regression model. Receiver operating characteristic (ROC) curve analysis was used to determine the value of serum IL-17A and IL-10 levels and in predicting HADAS. The prediction point was considered the point with the highest sensitivity and specificity. The level of significance was taken at *p* < 0.05.

## 3. Results

### 3.1. Symptom Scores

**Figure 1:**
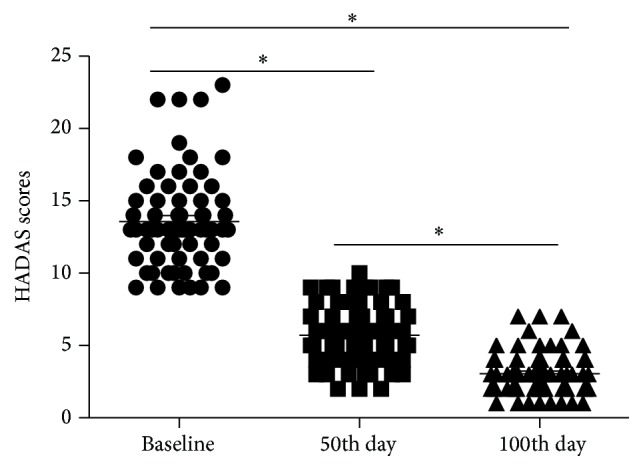
Scores of HADAS subjects. HADAS = high-altitude deacclimatization syndrome. The levels of HADAS scores of subjects tended to decrease as times went on. The dot plots show the levels of HADAS scores at baseline, 50th day, and 100th day. ^*∗*^ *p* < 0.05; the scores were significant different.

The HADAS scores were evaluated by physicians on the 3rd, 50th, and 100th days upon returning to low-altitude areas following 6 months of exposure to high altitudes and are shown in Figure 1.

The scores of subjects were 13.59 (0.41, 12.77–14.40) at baseline, 5.72 (0.25, 5.22–6.22) at the 50th day, and 3.06 (0.19, 2.69–3.43) at the 100th day, respectively. The differences in mean scores between baseline and the 50th day and 100th day were 7.87 (0.18, 7.41–8.32) (*p* < 0.001) and 10.52 (0.23, 9.54–11.09) (*p* < 0.001), respectively. The difference in mean scores between the 50th day and 100th day was 2.66 (0.10, 2.42–2.90) (*p* < 0.001). Thus, these data demonstrated that the HADAS scores decreased over time [6].

### 3.2. Serum Levels of SOD, MDA, IL-17A, IL-10, and TNF-*α* between HADAS and Control Groups

**Figure 2:**
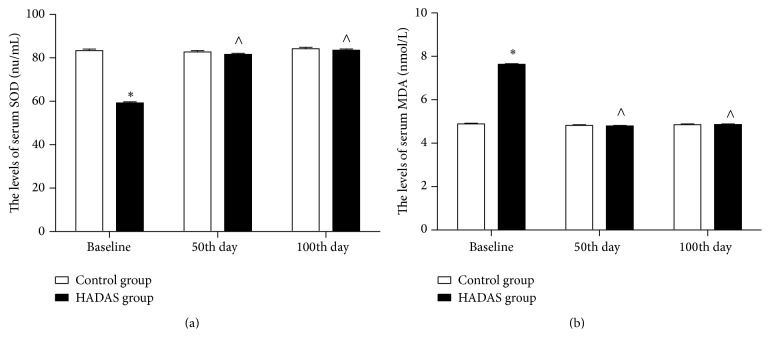
The serum SOD and MDA levels of subjects between HADAS and control groups. Data are presented as mean ± SE. SOD = superoxide dismutase; MDA = malondialdehyde. The serum SOD (a) and MDA (b) of subjects in both groups were assayed at baseline, 50th day, and 100 day. ^*∗*^ *p* < 0.05, relative to control group; ^∧^ *p* < 0.05, relative to baseline.

**Figure 3:**
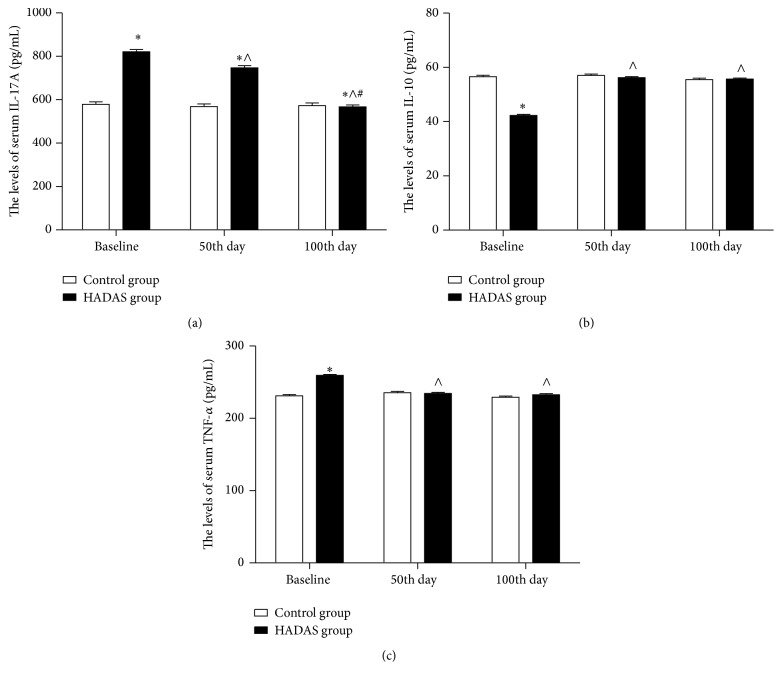
The serum IL-17A, IL-10, and TNF-*α* level of subjects between HADAS and control groups. Data are presented as mean ± SE. IL-17A = interleukin-17A, IL-10 = interleukin-10, and TNF-*α* = tumor necrosis factor-*α*. The serum IL-17A (a), IL-10 (b), and MDA (c) of subjects in both groups were assayed at baseline, 50th day, and 100 day. ^*∗*^ *p* < 0.05, relative to control group, ^∧^ *p* < 0.05, relative to baseline, and ^#^ *p* < 0.05, relative to the 50th day.

The serum concentrations of SOD, MDA, IL-17A, IL-10, and TNF-*α* were measured and compared between HADAS and control groups (Figures 2 and 3). At baseline, mean serum SOD and IL-10 levels were significantly lower in the HADAS group [serum SOD: 59.25 (0.52, 58.22–60.28) nu/mL versus 83.39 (0.67, 82.08–84.71) nu/mL, *p* < 0.001; serum IL-10: 42.29 (0.41, 41.47–43.10) pg/mL versus 56.55 (0.53, 55.51–57.59) pg/mL, *p* < 0.001]. Serum levels of SOD and IL-10 were not significantly different between HADAS and control groups at the 50th day and 100th day [serum SOD at the 50th day: 81.63 (0.52, 80.60–82.66) nu/mL versus 82.68 (0.67, 81.37–84.00) nu/mL, *p* = 0.21; serum IL-10 at the 50th day: 56.22 (0.41, 55.41–57.03) pg/mL versus 57.01 (0.53, 55.97–58.05) pg/mL, *p* = 0.24; serum SOD at the 100th day: 83.58 (0.52, 82.55–84.61) nu/mL versus 84.24 (0.67, 82.92–85.56) nu/mL, *p* = 0.44; serum IL-10 at the 100th day: 56.68 (0.41, 54.87–56.49) pg/mL versus 55.52 (0.53, 54.48–56.56) pg/mL, *p* = 0.81]. Moreover, serum SOD and IL-10 levels of HADAS subjects were lower at baseline than at the 50th and 100th days (*p* < 0.001).

Mean serum levels of MDA, IL-17A, and TNF-*α* were significantly higher in the HADAS group at baseline [serum MDA: 7.64 (0.03, 7.59–7.69) *μ*mol/mL versus 4.89 (0.04, 4.82–4.96) *μ*mol/mL, *p* < 0.001; serum IL-17A: 821.41 (9.52, 802.68–840.14) pg/mL versus 578.38 (12.17, 554.44–602.33) pg/mL, *p* < 0.001; serum TNF-*α*: 259.03 (1.48, 256.16–261.89) pg/mL versus 231.11 (1.86, 227.46–233.57) pg/mL, *p* < 0.001]. Compared with the control group, serum IL-17A was higher at the 50th day [747.36 (9.52, 728.63–766.09) pg/mL versus 568.58 (12.17, 544.63–592.52) pg/mL, *p* < 0.001]. IL-17A concentrations were not significantly different between HADAS and control groups at the 100th day [566.90, 9.52 (548.17–585.64) pg/mL versus 573.12, 12.17 (549.17–597.06) pg/mL, *p* = 0.69]. Serum MDA and TNF-*α* levels were not significantly different between HADAS and control groups at the 50 and 100th days [serum MDA at the 50th day: 4.79 (0.03, 4.74–4.85) *μ*mol/mL versus 4.819 (0.04, 4.75–4.89) *μ*mol/mL, *p* = 0.56; serum TNF-*α* at the 50th day: 232.19 (1.46, 229.32–235.05) pg/mL versus 229.91 (1.86, 226.24–233.57) pg/mL, *p* = 0.34; serum MDA levels at the 100th day: 4.86 (0.03, 4.81–4.92) *μ*mol/mL versus 4.85 (0.04, 4.78–4.92) *μ*mol/mL, *p* = 0.83; TNF-*α* levels at the 100th day: 232.19 (1.46, 229.32–235.05) pg/mL versus 229.91 (1.86, 226.24–233.57) pg/mL, *p* = 0.34]. In addition, serum MDA, IL-17A, and TNF-*α* levels of HADAS subjects were higher at baseline than at the 50th and 100th days (*p* < 0.001), and the IL-17A concentration at the 50th day was higher than at the 100th day (*p* < 0.001).

### 3.3. Correlation Analysis of Scores, SOD, MDA, IL-17A, IL-10, and TNF-*α* of HADAS Subjects at Baseline

To understand correlations of scores, SOD, MDA, IL-17A, IL-10, and TNF-*α* of HADAS subjects at baseline, all baseline data from HADAS subjects were subjected to correlation analysis (Table 2).

HADAS score was positively correlated with serum levels of IL-17A and TNF-*α* (*r* = 0.44, *p* < 0.001; *r* = 0.67, *p* < 0.001, resp.) and negatively correlated with serum levels of IL-10 (*r* = −0.56, *p* < 0.001). However, HADAS score was not correlated with serum SOD or MDA or age of HADAS subjects (*p* > 0.05).

Serum IL-17A level was negatively correlated with IL-10 and TNF-*α* levels (*r* = −0.33, *p* = 0.003; *r* = −0.33, *p* = 0.003, resp.). However, serum IL-17A level was not correlated with serum SOD or MDA or age of HADAS subjects (*p* > 0.05). It is interesting that serum IL-10 level was negatively correlated with TNF-*α* level and subject ages (*r* = −0.45, *p* < 0.001; *r* = −0.24, *p* = 0.03, resp.). Moreover, there was no correlation between IL-10 and SOD and MDA (*p* > 0.05).

### 3.4. Correlation Analysis of Scores, SOD, MDA, IL-17A, IL-10, and TNF-*α* Associated with HADAS Occurrence and Grading

Correlations of IL-17A, TNF-*α*, and IL-10 with HADAS occurrence and severity were analyzed via logistic regression. These data showed that serum IL-17A level was associated with HADAS severity (*p* < 0.05) (Table 3), and serum IL-17A, TNF-*α*, and IL-10 levels were associated with HADAS occurrence (*p* < 0.01). In addition, SOD and MDA levels and subject ages were not significantly associated with HADAS occurrence (*p* = 0.984, *p* = 0.994, and *p* = 0.984, resp.). Multivariate logistic regression analysis showed that IL-17A level (OR = 1.025, *p* = 0.044) and IL-10 level (OR = 0.681, *p* = 0.017) were predictive of HADAS (Table 4).

**Figure 4:**
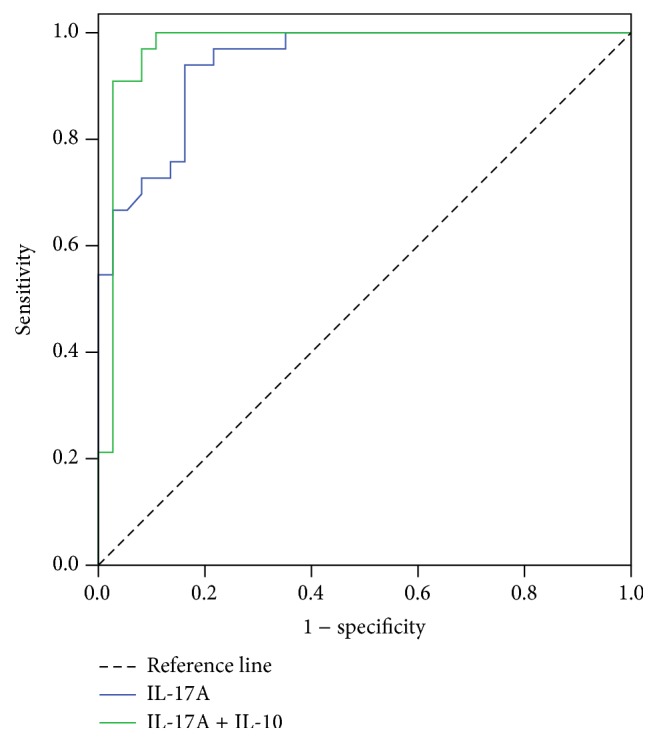
ROC curve analysis of the prediction of HADAS based on the serum IL-17A and combination of serum IL-17A and IL-10 levels. ROC curve analysis showing the performance of the biomarker IL-17A alone (blue) and a combination of IL-17A and IL-10 (green) in predicting the development of HADAS on the baseline. HADAS = high-altitude deacclimatization syndrome, IL-17A = interleukin-17A, and IL-10 = interleukin-10.

To study further the role of serum levels of IL-17A and IL-10 in HADAS, ROC curve analysis was used (Figure 4). These data demonstrated that serum IL-17A predicted HADAS with sensitivity of 93.9% and specificity of 77.7% [AUC = 0.941 ± 0.025 (SE), 95% CI: 0.892–0.991, *p* < 0.001]. These data demonstrated that a serum IL-17A level > 726.41 pg/mL predicted HADAS with sensitivity of 83.8% and specificity of 93.9% [AUC = 0.941 ± 0.025 (SE), 95% CI: 0.892–0.991, *p* < 0.001]. A serum IL-10 level < 48.76 pg/mL predicted HADAS with specificity of 89.2% and sensitivity of 93.9% [AUC = 0.973 ± 0.022 (SE), 95% CI: 0.000–1.000, *p* < 0.001]. It is interesting that combining IL-17A and IL-10 levels provided an additional benefit for predicting HADAS with specificity of 97.3% and sensitivity of 97.0% [AUC = 0.984 ± 0.016 (SE), 95% CI: 0.000–1.000, *p* < 0.001]. The findings indicated that the combination of serum IL-17A and IL-10 levels was a better diagnostic predictor of HADAS than serum IL-17A or IL-10 levels alone.

## 4. Discussion

Our data showed that the HADAS group had a significantly higher serum level of IL-17A compared with the control group at baseline and the 50th day and serum levels of IL-17A and HADAS score decreased over time in the HADAS group. Furthermore, serum levels of MDA and TNF-*α* were significantly higher and SOD and IL-10 levels were lower in the HADAS group than in the control group at baseline. Serum levels of IL-17A, IL-10, and TNF-*α* were significantly correlated with HADAS score. Serum IL-17A was correlated with HADAS severity, and serum levels of IL-17A and IL-10 were significantly correlated with HADAS occurrence rate. Thus, serum levels of IL-17A and IL-10 could be novel diagnostic predictors of HADAS.

H/R-mediated oxidative stress is involved in occurrence of HADAS. SOD is an antioxidant that reduces oxidative stress and protects tissues from damage induced by ROS [23]. Serum MDA is considered a marker of oxidative damage [24]. In our study, suppression of serum SOD and elevation of MDA levels was observed in HADAS subjects at baseline. Thus, these data indicate that the HADAS subjects had experienced oxidative stress. Serum SOD and MDA levels were not significantly different between the HADAS and control groups at the 50th and 100th days. This suggests that the levels of oxidative stress in HADAS subjects returned to normal levels. In addition, serum levels of SOD and MDA in HADAS subjects were not correlated with HADAS scores, severity, or occurrence rate at baseline. This could be because baseline serum levels of SOD and MDA reflected the very early stages of oxidative stress and antioxidant status in HADAS subjects, and they were indirectly involved in the appearance of symptoms.

H/R-mediated oxidative stress could induce release of mediators of inflammation and activate many signaling pathways, which result in cellular apoptosis and tissue damage [21, 25–29]. In our study, levels of proinflammatory mediators, TNF-*α* and IL-17A, were elevated, and levels of anti-inflammatory factor IL-10 were reduced at baseline in the HADAS group. This data suggests that an H/R-induced inflammatory response was involved in the occurrence of HADAS. Moreover, serum levels of IL-17A decreased over time in the HADAS group and were not different between HADAS and control groups at 100 d. Similar to serum levels of SOD and MDA, serum levels of IL-10 and TNF-*α* were not significantly different from the control group at the 50th and 100th days. These data suggest that the H/R-induced inflammatory response decreased with time.

Cytokine IL-17A is a member of the IL-17 family, and it is the hallmark cytokine of Th17 cells. Serum levels of IL-17A are elevated in several chronic inflammatory diseases [30–32], and IL-17A plays an important role in regulating inflammatory mediators and the inflammatory response. The mechanism of IL-17A in inflammation involves mediating recruitment of neutrophils to sites of inflammation and activating a number of proinflammatory chemokines and matrix metalloproteases [22]. Recently, the role of IL-17A in H/R or ischemia/reperfusion (I/R) has been explored. Barry and colleagues showed that elevation of IL-17A played a fundamental role in inflammation and apoptotic response in myocardial I/R injury [33]. Similarly, another study demonstrated that levels of serum IL-17A were increased by neutrophils and CD4^+^ T cells produced following experimental I/R injury in mice [34]. Friedrich and colleagues [35] showed that IL-17A strongly induced TNF-*α* expression in inflammatory bowel disease. Xue and colleagues [36] showed that increased IL-17A not only enhanced production of proinflammatory cytokines but also impaired production of anti-inflammatory factors such as IL-10, resulting in renal tissue injury after I/R. Lee et al. [37] demonstrated that IL-17A played a critical role in intestinal, renal, and liver injury after I/R, and IL-17A knockout or inactivation significantly alleviated intestinal I/R injury and subsequent liver and kidney dysfunction. Based on the above research, we speculated that IL-17A may play a more important role than other inflammatory mediators, such as IL-10 and TNF-*α*, in the occurrence of HADAS.

Exploring biomarkers for HADAS is very necessary. The diagnostic and scoring criteria of HADAS [6] included the essential diagnostic criteria, auxiliary diagnostic criteria, and symptom scores of HADAS. This was a complex diagnostic system, especially for HADAS symptom scores. Twenty-one symptoms had to be evaluated in scoring of HADAS symptoms by physicians and researchers. Evaluation of HADAS symptoms scores was a very heavy workload for physicians. Furthermore, symptom severity of HADAS subjects was based on subjective feelings, which resulted in some error for HADAS symptom scores. The present findings showed that serum levels of IL-17A, TNF-*α*, and IL-10 were significantly correlated with the HADAS score. It is interesting that IL-17A level was significantly associated not only with severity of HADAS, but also with HADAS occurrence based on multivariate logistical regression analysis. Serum level of IL-10 also correlated with occurrence of HADAS, and there was no correlation between IL-10 level and HADAS severity. This indicated that the serum level of IL-17A was a better independent predictor of HADAS than IL-10. In addition, ROC indicated that the combination of serum levels for IL-17A and IL-10 was a better predictor of HADAS occurrence than serum levels of IL-17A or IL-10 alone. These data suggest that a combination of serum levels for IL-17A and IL-10 may be a novel diagnostic predictor of HADAS.

Our study has some limitations. First, all of the subjects were male, and the majority of subjects were an average of 25 years old. Second, the spans between evaluation time points were too long to evaluate some parameters such as SOD, MDA, IL-10, and TNF-*α*, which had already returned to normal levels in the HADAS group. All of these factors may have introduced bias into the results.

In conclusion, H/R-mediated oxidative stress and an inflammatory response are involved in occurrence of HADAS, and IL-17A level could be a novel predictive index of HADAS.

## Figures and Tables

**Table 1:** Baseline characteristics of subjects at randomization according to the study group.

	HADAS group (*n* = 67)	Control group (*n* = 41)	*p* value
Demographic characteristics			
Age, years (SD, range)	25.1 (7.6, 18–35)	24.8 (8.1, 18–34)	>0.05
Race, Han (%)	67 (100%)	41 (100%)	>0.05
Sex, male (%)	67 (100%)	41 (100%)	>0.05
Symptom score (SE, 95% CI)	13.58 (0.41, 12.77–14.39)		
Severity of HADAS			
Moderate reaction (%)	15 (22.39%)		
Mild reaction (%)	52 (77.61%)		

The data indication (%) or the mean [(SD; range) or (SE, 95% CI)]. Symptom score = high-altitude deacclimatization syndrome (HADAS) scores.

**Table 2:** Correlation analysis of scores, SOD, MDA, IL-17A, IL-10, and TNF-*α* of HADAS subjects on the baseline.

Variables	Scores		SOD		MDA		IL-17A		IL-10		TNF-*α*	
	*r*	*p* value	*r*	*p* value	*r*	*p* value	*r*	*p* value	*r*	*p* value	*r*	*p* value
Scores	—	—	−0.07	0.29	0.11	0.20	0.44	0.000^*∗*^	−0.56	0.000^*∗*^	0.67	0.000^*∗*^
SOD	−0.07	0.29	—	—	0.04	0.39	−0.004	0.49	−0.12	0.16	0.06	0.31
MDA	0.11	0.20	0.04	0.39	—	—	0.08	0.26	−0.08	0.27	0.10	0.22
IL-17A	0.44	0.000^*∗*^	−0.004	0.49	0.08	0.26	—	—	−0.33	0.003^*∗*^	−0.33	0.003^*∗*^
IL-10	−0.56	0.000^*∗*^	−0.12	0.16	−0.08	0.27	−0.33	0.003^*∗*^	—	—	−0.45	0.000^*∗*^
TNF-*α*	0.67	0.000^*∗*^	0.06	0.31	0.10	0.22	−0.33	0.003^*∗*^	−0.45	0.000^*∗*^	—	—
Age	0.08	0.27	−0.14	0.13	0.25	0.02^*∗*^	0.03	0.40	−0.24	0.03^*∗*^	0.11	0.18

^*∗*^A significant correlation (*p* < 0.05).

**Table 3:** Logistic regression analysis of the serum SOD, MDA, IL-17A, TNF-*α*, and IL-10 associated with HADAS severity.

Variable	Univariate		Multivariate	
	OR (95% CI)	*p* value	OR (95% CI)	*p* value
SOD	0.951 (0.868–1.043)	0.284	0.928 (0.819–1.053)	0.247
MDA	2.789 (0.468–16.636)	0.260	2.968 (0.251–35.144)	0.388
IL-17A	1.064 (1.023–1.107)	0.002^*∗*^	1.052 (1.012–1.093)	0.010^*∗*^
TNF-*α*	1.078 (1.025–1.133)	0.003^*∗*^	1.067 (0.989–1.150)	0.095
IL-10	0.702 (0.559–0.882)	0.002^*∗*^	0.854 (0.622–1.172)	0.328

^*∗*^ *p* < 0.05 is considered significant for statistical analyses.

**Table 4:** Logistic regression analysis of the serum IL-17A, TNF-*α*, and IL-10 associated with HADAS occurrence.

Variable	Univariate		Multivariate	
	OR (95% CI)	*p* value	OR (95% CI)	*p* value
IL-17A	1.033 (1.018–1.047)	0.000^*∗*^	1.029 (1.003–1.056)	0.030^*∗*^
TNF-*α*	1.081 (1.041–1.122)	0.000^*∗*^	1.087 (0.989–1.195)	0.083
IL-10	0.594 (0.466–0.758)	0.000^*∗*^	0.688 (0.505–0.938)	0.018^*∗*^

^*∗*^ *p* < 0.05 is considered significant for statistical analyses.

## Abbreviations

HADAS: High-altitude deacclimatization syndrome
H/R: Hypoxia/reoxygenation
I/R: Ischemia/reperfusion
IL-17A: Interleukin-17A
IL-10: Interleukin-10
TNF-*α*: Tumor necrosis factor-*α*
SOD: Superoxide dismutase
MDA: Malondialdehyde
ROC: Receiver operating characteristic.
